# Sucrose and starch intake contribute to reduced alveolar bone height in a rodent model of naturally occurring periodontitis

**DOI:** 10.1371/journal.pone.0212796

**Published:** 2019-03-13

**Authors:** Juliano Morimoto, Alistair Senior, Kate Ruiz, Jibran A. Wali, Tamara Pulpitel, Samantha M. Solon-Biet, Victoria C. Cogger, David Raubenheimer, David G. Le Couteur, Stephen J. Simpson, Joerg Eberhard

**Affiliations:** 1 Charles Perkins Centre, Camperdown, New South Wales, Australia; 2 Programa de Pós-Graduação em Ecologia e Conservação, Federal University of Paraná, Curitiba, Brazil; 3 Department of Biological Sciences, Macquarie University, Sydney, New South Wales, Australia; 4 The University of Sydney, School of Mathematics and Statistics, Camperdown, New South Wales, Australia; 5 The University of Sydney, School of Life and Environmental Sciences, Camperdown, New South Wales, Australia; 6 The University of Sydney Dental School, Faculty of Health and Medicine, The University of Sydney, Sydney, New South Wales, Australia; 7 Centre for Education and Research on Ageing, Concord, New South Wales, Australia; 8 Concord Repatriation General Hospital, Concord, New South Wales, Australia; University of Illinois, UNITED STATES

## Abstract

While there is a burgeoning interest in the effects of nutrition on systemic inflammatory diseases, how dietary macronutrient balance impacts local chronic inflammatory diseases in the mouth has been largely overlooked. Here, we used the Geometric Framework for Nutrition to test how the amounts of dietary macronutrients and their interactions, as well as carbohydrate type (starch vs sucrose vs resistant starch) influenced periodontitis-associated alveolar bone height in mice. Increasing intake of carbohydrates reduced alveolar bone height, while dietary protein had no effect. Whether carbohydrate came from sugar or starch did not influence the extent of alveolar bone height. In summary, the amount of carbohydrate in the diet modulated periodontitis-associated alveolar bone height independent of the source of carbohydrates.

## Introduction

Gingivitis and periodontitis belong to a spectrum of inflammatory periodontal diseases caused by bacterial accumulation that damage the tooth-supporting tissues. Irreversible high-level inflammatory periodontitis, if left untreated, leads to tooth loss [1]. The prevalence of moderate to severe periodontitis in Western populations is approximately 50%, while the prevalence of gingivitis is as high as 62–94% [2, 3]. In addition to local inflammatory processes periodontitis and gingivitis increase susceptibility to many systemic diseases including cardiovascular and respiratory diseases, immune deficiencies and type 2 diabetes mellitus [4].

It has been well established that dietary sugar and fermentable carbohydrates lead to the initiation and progression of dental decay [5]. In a series of studies, beneficial effects of dairy products [6], probiotics [7, 8] or poly-unsaturated fatty acids [9] on periodontal disease have been demonstrated. In terms of macronutrients (protein, carbohydrates and fat) some studies have shown that a carbohydrate-rich diet increases the risk of inflammation [10, 11], while a ‘Paleolithic’ diet, which is high in protein, decreases gingival bleeding [12]. Findings from a series of animal experiments in the 1970s led investigators to report that a carbohydrate-free diet “prevents the initiation of periodontal lesions in the soft tissues” [13]. However, restriction of dietary carbohydrates in humans is impractical and may be undesirable because of the potential beneficial effects dietary carbohydrates, particularly those with a low glycemic index, on late life cardiometabolic health [14].

The Geometric Framework for Nutrition (GFN) provides a means to explore whether and how carbohydrates and other nutrients interact in their impacts on oral health. The GFN is a state-space approach that has been successfully applied to assess the integrated effects of nutrition in humans and a range of other organisms [15, 16]. Here we aimed to disentangle the effects of macronutrient intake and type of carbohydrate in the diet on alveolar bone height measured as the distance between the cemento-enamel junction (CEJ) and alveolar bone crest (ABC) using the rodent model of naturally occurring periodontitis developed by Liang et al [17] which contrasts to the majority of other rodent studies that induce periodontitis by the artificial application of human pathogens to the oral cavity. This is the first application of the GFN to oral health.

## Methods

### Mice

Male C57BL/6 mice (n = 300; 8 weeks old) were housed in groups of four animals/cage and maintained on 12hr day/light cycle. The mice were fed ad libitum one of 15 isocaloric low-protein-high carbohydrate diets composed of differing percentages of protein (5, 10 and 15% of total net metabolisable energy) and carbohydrate (75, 70 and 65%) while fat was maintained at 20% in all diets. These diets were formulated based on the ingredients in the AIN93G standard rodent diet [18] and were manufactured by Specialty Feeds (Glen Forrest, Australia). The AIN93G standard diet provides about 19% energy from protein, so all the diets were low in protein content compared to the standard chow. The aim of our study was to investigate how these low protein-high carbohydrate diets affect dental health, if their effects are dependent on the type of dietary carbohydrate, and how increasing protein content (within the low protein bracket) modulates carbohydrate effects. To investigate the effects of carbohydrate type on oral health, diets were further distinguished by systematically changing the sucrose-starch ratios (20/80, 35/65, 50/50, 65/35, 80/20) in their carbohydrate component (Table 1). We used starch [19] and sucrose as they are the two major types of carbohydrate in human diet. The starch used in these diets was sourced from wheat. This feeding array allows examination of the impact of each individual food component (protein, sucrose and starch) and their interactions using the GFN methodology. We also maintained 40 mice on two diets (n = 20 per diet) containing 10% protein where native wheat starch was replaced by a high fibre ‘resistant starch’ (Crisp Film^™^ starch, Ingredion, Westchester, IL), which is relatively resistant to digestion by amylase while keeping the diets isocaloric. Mice were maintained on experimental diets for 18–19 weeks and were euthanised with 75mg/kg intra-peritoneal pentobarbital for tissue collection. All animal procedures involved in this study were approved by the University of Sydney animal ethics committee (protocol# 2015/881) and all methods were performed in accordance with the Australian Code of Practice for the Care and Use of Animals for Scientific Purposes.

**Table 1:** Composition of diets by net metabolizable energy (%)—14.3 kJ/gram.

Carbohydrate composition	Protein 5% + Fat 20% + Carbohydrate 75%					Protein 10% + Fat 20% + Carbohydrate 70%					Protein 15% + Fat 20% + Carbohydrate 65%				
***Sucrose %***	20	35	50	65	80	20	35	50	65	80	20	35	50	65	80
**+**	+	+	+	+	+	+	+	+	+	+	+	+	+	+	+
***Starch %***	80	65	50	35	20	80	65	50	35	20	80	65	50	35	20
***Sucrose %***							35			80					
**+**	X	X	X	X	X	X	+	X	X	+	X	X	X	X	X
***Resistant Starch %***							65			20					

### Measurement of food intake

Food intake was measured as described previously [20]. Briefly, a weighed amount of food was added to the hoppers of mouse cages at the start of each week, and the food left in the hoppers after 7 days was measured. In addition, all the particles of food that fell from the hoppers into the bedding were carefully collected and weighed. This food intake measurement was done for at least five consecutive weeks, and from the difference in food weight at the start and end of the week, the average food intake per mouse per cage was calculated.

### Measurement of the alveolar bone height represented as the distance between CEJ and ABC

**Fig 1:**
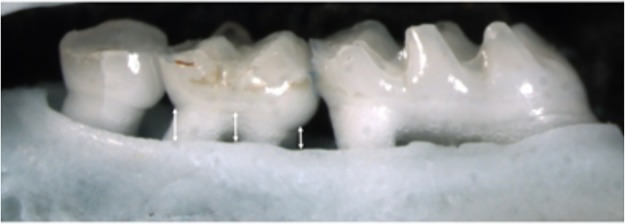
The distance between the CEA and the ABC as a measure of bone height. The distance between the cemento-enamel junction and the most coronal part of the surrounding bone was measured at three sites of the middle molar tooth.

We manually dissected the maxilla of mice and, to remove the remaining tissue, a solution of 1% NaOH was added and boiled for 4 min at 100°C, after which the solution was disposed. Jaws were stained to highlight the cementum-enamel junction with a solution of 0.04% methylene blue for 1 min. Samples were dried at 65°C for 12h, before taking standardised photographs. As a measure of alveolar bone height, we measured the distance between the CEJ and ABC, where larger measures of distance correspond to a lower bone height and consequently poorer oral health. The distance between the CEJ and ABC was measured in millimetres at 3 positions of the buccal region of the second molar on both sides of the maxilla (Fig 1). All measurements were performed using ImageJ.

### IL-1ß measurement in gingival tissues

Gingival tissues were isolated from mice jaws and homogenised (Tissue homogenizer, Qiagen, Hilden, Germany) for 1 min while immersing in RIPA buffer. The solution was sonicated for 1 min and centrifuged for 10 min at 16000g and 4°C. The protein concentration in the supernatant was determined in BCA solution (B9843, Sigma-Aldrich, MO) using a microplate reader at a wavelength of 532 nm (Infinite M1000 pro, Tecan, Männedorf, Switzerland). IL-1ß concentration was determined by ELISA (AVIVA Systems Biology, CA) according to the manufacturer’s instructions. The results were normalized for the protein content of each sample and are expressed as mean ± SD for three experiments.

### Statistical analyses

Data were analysed, and surfaces depicting the effects of macronutrient intakes on CEJ-ABC distance generated, using two generalised additive mixed models (GAMMs) of the Gaussian (identity-link function). GAMMs were more appropriate than linear models as previous studies applying the GFN have shown that macronutrient intakes can be associated with complex non-linear effects of nutrient intake on health in mice [20]. In all models, the response variable was CEJ-ABC distance (a negative correlate of bone height) of a tooth, and the identity of the animal from which the measurement was fitted as a random effect (to correct for non-independence among measurements taken from teeth from the same animal) [21]. To test for the effect of carbohydrate and protein intake on alveolar bone height, we included daily intakes (KJ/mouse/day) of protein and total carbohydrates, and their interaction, as fixed predictors, fitted with thin-plate smoothing. To test for the effect of starch and sucrose intake, we fitted a similar model but decomposed total carbohydrates to starch and sucrose intakes, which were fitted along with and protein intake, and their two-way interactions, as fixed predictors. The effects of resistant starch were explored using linear mixed-models (LMMs), fitting the amount of bone height in the two diets with resistant starch and that in the diets with equivalent composition and non-resistant starch as a response. The predictors were the identity of the animals as a random effect, and two categorical predictors as fixed effects denoting the starch content of the diet and whether the starch was resistant fitted with interaction. GAMMs were implemented using the ‘gamm’ function in the package *mgcv* [22–24] in the statistical programming environment *R* (version 3.4.0) [25], and LMMs with the ‘lmer’ function in the package *lme4* [26]. *p* values < 0.05 were considered statistically significant. Finally, to test for an association between the carbohydrate content of the diet and *IL-1ß* measurements for each mouse using a Pearson’s correlation coefficient.

## Results

### High carbohydrate intake induces a reduction in bone height

**Fig 2:**
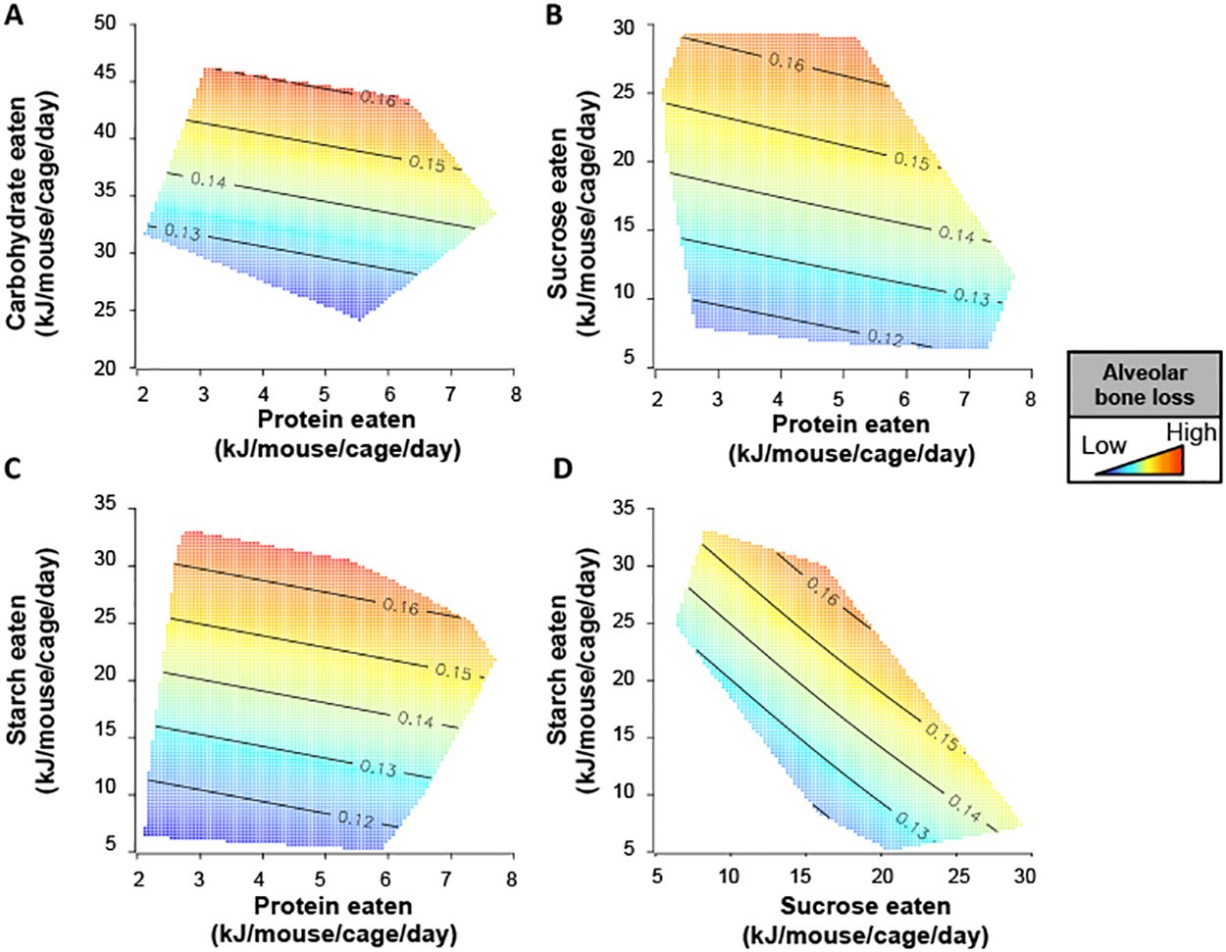
Sucrose and starch intake increase the distance between CEJ and ABC, corresponding to reduced bone height in mice. Surfaces showing predicted effect of macronutrient intake on CEJ-ABC distance as given by generalized additive mixed models (GAMMs) with thin-plate splines. The results of two GAMMs are shown; one fitting only protein and carbohydrate (A), and another fitting protein, sucrose and starch (B through D). In (B through D), predicted effects assumes intake of the third macronutrient (i.e. that not displayed on the x and y axes) is held constant at the experimentally observed median. In all surfaces, red indicates the highest value, while blue indicates the lowest value.

There were substantial differences among diets in mean CEJ-ABC distance (Table 2; ANOVA for effect of diet, d.f. 16, 1407, F = 2.827, p < 0.001). Increasing intake of total carbohydrates is associated with a linear increase in the CEJ-ABC distance in mice (Table 3, Fig 2A). On the other hand, protein intake had no effect on CEJ-ABC distance (Table 3, Fig 2A). There was no evidence for an interaction between the effects of intake of protein and carbohydrates on CEJ-ABC distance, and consequently bone height (Table 3, Fig 2A).

**Table 2:** Means and SD (mm) for bone loss measured for different diets and sucrose/starch content included in the study.

Diet	n	mean	SD	P/C/F (%)	Sucrose/starch (%)
**G-1/S-1**	62	0.156	0.05	5/75/20	20/80
**G-1/S-2**	105	0.14	0.055	5/75/20	35/65
**G-1/S-3**	88	0.153	0.049	5/75/20	50/50
**G-1/S-4**	105	0.132	0.045	5/75/20	65/35
**G-1/S-5**	47	0.128	0.046	5/75/20	80/20
**G-2/S-1**	117	0.136	0.047	10/70/20	20/80
**G-2/S-2**	106	0.145	0.062	10/70/20	35/65
**G-2/S-2R**	93	0.172	0.07	10/70/20	35/65
**G-2/S-3**	84	0.144	0.051	10/70/20	50/50
**G-2/S-4**	107	0.131	0.05	10/70/20	65/35
**G-2/S-5**	93	0.125	0.047	10/70/20	80/20
**G-2/S-5R**	96	0.152	0.051	10/70/20	80/20
**G-3/S-1**	12	0.121	0.03	15/65/20	20/80
**G-3/S-2**	103	0.132	0.053	15/65/20	35/65
**G-3/S-3**	93	0.123	0.043	15/65/20	50/50
**G-3/S-4**	107	0.143	0.052	15/65/20	65/35
**G-3/S-5**	6	0.078	0.031	15/65/20	80/20

**Table 3:** Coefficients (Coef.) of smoothed terms from generalised additive mixed models (GAMM) of CEJ-ABC distance (negative associated with alveolar bone height) as a function of protein (P), carbohydrate (C), sucrose (Su) and starch (St) intake. d.f. = degrees of freedom. In both models, the estimated residual and among-mouse standard deviation was 0.04. **Bold**—p < 0.05.

Model	Coef.	Error d.f.	Reference d.f.	F-value	*p-value*
1: Protein and Carbohydrate	P	1.00	1	1.12	0.29
	C	1.00	1	10.13	**< 0.01**
	P:C	3.29 × 10^−9^	27	0.00	0.50
2: Protein, Sucrose and Starch	P	1.00	1.00	1.07	0.30
	Su	1.22	1.22	4.01	**0.03**
	St	1.00	1.00	9.53	**< 0.01**
	P:Su	1.28 × 10^−6^	27.00	0.00	0.26
	P:St	2.97 × 10^−8^	27.00	0.00	1.00
	Su:St	5.63 × 10^−6^	4.00	0.00	0.85

Together, these results corroborate the established premise that dietary carbohydrate is a driver of periodontitis, and demonstrates its effect is independent of the other macronutrients, specifically dietary protein.

When the source of carbohydrates was analysed, we found that increasing the intake of either sucrose or starch led to significant increase in the distance between CEJ and ABC (Table 1, Fig 2B, 2C and 2D). The magnitude of the effect of these two carbohydrate sources were statistically indistinguishable (Fig 2D). Putting the results of these analyses in biological context, doubling the daily intake of either sucrose and/or starch was associated with a 17% increase in the CEJ—ABC distance at 18–19 months of age in mice.

### Resistant starch does not protect against a reduced alveolar bone height in mice

We assessed whether resistant starch had any effect on oral health and diet-induced increase of the distance between CEJ and ABC. LMMs estimated that the distance between CEJ and ABC was slightly larger in groups of animals fed resistant starch diets relative to equivalent diets with non-resistant starch, although these differences were not statistically significant (Table 4). The results indicate that resistant starch does not protect mice against a reduction of alveolar bone height.

**Table 4:** Coefficients (Coef.) from linear mixed models (LMMs) of the estimated (Est.) effects of starch content (20% vs 65%) and starch type (non-resistant or resistant) on distance between CEJ and ABC in mice, and associated standard errors (SE). d.f. = degrees of freedom. The estimated among-mouse and residual standard deviation was 0.046 and 0.014, respectively. **Bold**—p < 0.05.

Coef.	Est.	SE	d.f.	t-value	*p-value*
***Intercept***	0.126	0.012	62.76	10.62	**<0.001**
***% Starch***	0.019	0.017	63.80	1.153	0.253
***Type***	0.025	0.017	63.81	1.499	0.139
***% Starch*: *Type***	0.002	0.024	64.08	0.076	0.940

### Interleukin-1 concentrations in gingival tissues

The mean concentration of IL-1ß in gingival tissues was 1.046 ± 0.565 ng/mg total protein (min 0.295 ng/mg; max 2.952 ng/mg). There was an association between dietary carbohydrate content and IL-1ß, although the effect was not statistically significant (*p* = 0.08).

## Discussion

Our study confirms that dietary carbohydrate intake has a negative impact on periodontal health in a mouse model of naturally occurring periodontitis. In addition, we have shown the effect of carbohydrates is independent of dietary protein or the type of carbohydrate—sucrose, wheat starch and resistant starch all have the same negative effects on alveolar bone height, measured as the distance between the CEJ and ABC in this study. The quantity of carbohydrate eaten—rather than the type of carbohydrate (among those tested)—influences oral health in mice. This observation is in agreement with studies that demonstrated that starch has the potential to be cariogenic, mediated by elongated retention times in the oral cavity compared to sucrose [27]. We varied the protein-to-carbohydrate ratio in the diet because our recent studies have shown that the interaction between carbohydrate and protein in the diet influences multiple aspects of physiology and health [20]. While the cariogenic potential of carbohydrates is solely related to its local effects on the tooth surfaces, it is possible that dietary components modulate periodontal disease by shaping the systemic immune response. This hypothesis has been supported by animal experiments which demonstrated that the administration of probiotics positively affects periodontal health in mice, irrespectively of the administration by lavage or gavage [8]. The systemic modulation of the periodontal immune response is in accordance with the concept that periodontitis arises from an inappropriate inflammatory reaction to the normal microbiota [28].

Recent studies have shown that addition of resistant starch to diet improves the metabolic profile of mice and provides protection from certain chronic inflammatory disorders including inflammatory colitis. This is mediated by beneficial changes in gut microbiome and increased circulating concentration of short chain fatty acids [29]. However, dietary intake of resistant starch did not protect mice from a reduction of alveolar bone height in this rodent model.

A major risk factor for periodontitis is ageing [30]. It is therefore important to disentangle the indirect effects of diet in our study on periodontitis via its effect on ageing from the direct effects of diet on the oral cavity and teeth. We reported that ad libitum-fed diets that are low in protein and higher in carbohydrate are associated with a slower rate of ageing and longer lifespan [20]. Alveolar bone height observed in our study was lowest on those diets that optimized age-related health and lifespan. This suggests that the effects of dietary carbohydrates on oral health are mediated by their direct effect on the oral cavity, rather than indirectly via their effects on systemic health.

Induction of experimental periodontitis in mice is typically achieved by oral lavage with human periodontal pathogens and measurable changes in the distance between CEJ and ABC, and respectively bone height requires several weeks following oral infection [8, 31]. However, mice do develop naturally occurring periodontitis as they age that is induced by their own oral flora [17]. We evaluated periodontitis by measuring alveolar bone height—calculated as the average distance between the cementum-enamel junction and alveolar bone crest. A reduced height of alveolar bone is the main correlate of periodontitis in humans and is measured in the same way in mice in our study. The reason that sham infected mice do not develop obvious signs of periodontitis is likely due to their young age (usually used when 8-12-week-old), consistent with the fact that periodontitis is associated with advanced age [32]. When the immune status is genetically altered, young mice develop periodontitis even when they are not inoculated with human pathogens [33]. In both cases, induction of inflammation and periodontal bone loss is prevented by antibiotics, thus further confirming indigenous bacterial involvement in the disease. Liang et al. [17] also provided evidence for the inflammatory nature of natural occurring periodontitis in mice and that attrition to dentition is not a factor involved in a reduction of bone height measurements in this model.

The levels of IL-1ß suggest that the mice in our study were not free from periodontitis. Trombone et al. reported a IL-1ß concentration of 0.08 ng/mg 45 days after infection with *A*. *actinomycetemcomitans*, while no IL-1ß was detected in control animals [33]. Napimoga et al. reported values of 0.013 ng/mg IL-1ß in infected gingival tissues and 0.06 ng/mg IL-1ß in sham infected animals [34] and Lima et al. reported 0.05 ng/mg total protein IL-1ß in the gingival tissues 60 days after infection with *A*. *actinomycetemcomitans* [35]. The high concentrations of 1.046 ± 0.565 ng/mg total protein measured in our study are indicative of the presence of active periodontitis in older mice maintained on diets for their lifetime.

## Conclusion

In summary, this is the first study to explore macronutrient composition and types of carbohydrate on the development of natural occurring periodontitis in older animals. There was a positive correlation between a reduced bone height and the intake of dietary carbohydrate, whether in the form of sucrose, wheat starch, or resistant starch.

## References

[1] Pihlstrom BL, Michalowicz BS, Johnson NW. Periodontal diseases. Lancet. 2005;366(9499):1809–20. Epub 2005/11/22. 10.1016/S0140-6736(05)67728-8 . 16298220

[2] Li Y, Lee S, Hujoel P, Su M, Zhang W, Kim J, et al Prevalence and severity of gingivitis in American adults. Am J Dent. 2010;23(1):9–13. Epub 2010/05/05. . 20437720

[3] Ericsson JS, Abrahamsson KH, Ostberg AL, Hellstrom MK, Jonsson K, Wennstrom JL. Periodontal health status in Swedish adolescents: an epidemiological, cross-sectional study. Swed Dent J. 2009;33(3):131–9. Epub 2009/12/10. . 19994563

[4] Linden GJ, Herzberg MC, Working group 4 of joint EFPAAPw. Periodontitis and systemic diseases: a record of discussions of working group 4 of the Joint EFP/AAP Workshop on Periodontitis and Systemic Diseases. J Clin Periodontol. 2013;40 Suppl 14:S20–3. Epub 2013/05/03. 10.1111/jcpe.12091 . 23627330

[5] Pitts NB, Zero DT, Marsh PD, Ekstrand K, Weintraub JA, Ramos-Gomez F, et al Dental caries. Nat Rev Dis Primers. 2017;3:1–16. ARTN 17030 10.1038/nrdp.2017.30 28540937

[6] Adegboye AR, Christensen LB, Holm-Pedersen P, Avlund K, Boucher BJ, Heitmann BL. Intake of dairy products in relation to periodontitis in older Danish adults. Nutrients. 2012;4(9):1219–29. 10.3390/nu4091219 23112910 PMC3475232

[7] Slawik S, Staufenbiel I, Schilke R, Nicksch S, Weinspach K, Stiesch M, et al Probiotics affect the clinical inflammatory parameters of experimental gingivitis in humans. Eur J Clin Nutr. 2011;65(7):857–63. Epub 2011/03/31. 10.1038/ejcn.2011.45 . 21448219

[8] Gatej SM, Marino V, Bright R, Fitzsimmons TR, Gully N, Zilm P, et al Probiotic Lactobacillus rhamnosus GG prevents alveolar bone loss in a mouse model of experimental periodontitis. J Clin Periodontol. 2017 10.1111/jcpe.12838 . 29121411

[9] Eberhard J, Heilmann F, Acil Y, Albers HK, Jepsen S. Local application of n-3 or n-6 polyunsaturated fatty acids in the treatment of human experimental gingivitis. J Clin Periodontol. 2002;29(4):364–9. . 11966935 10.1034/j.1600-051x.2002.290413.x

[10] Hujoel P. Dietary carbohydrates and dental-systemic diseases. J Dent Res. 2009;88(6):490–502. Epub 2009/07/10. 10.1177/0022034509337700 . 19587153

[11] Woelber JP, Bremer K, Vach K, Konig D, Hellwig E, Ratka-Kruger P, et al An oral health optimized diet can reduce gingival and periodontal inflammation in humans—a randomized controlled pilot study. BMC Oral Health. 2016;17(1):28 Epub 2016/07/28. 10.1186/s12903-016-0257-1 . 27460471 PMC4962497

[12] Baumgartner S, Imfeld T, Schicht O, Rath C, Persson RE, Persson GR. The impact of the stone age diet on gingival conditions in the absence of oral hygiene. J Periodontol. 2009;80(5):759–68. Epub 2009/05/02. 10.1902/jop.2009.080376 . 19405829

[13] Shaw JH, Griffiths D. Development and post-developmental influences on incidence of experimental dental caries resulting from dietary supplementation by various elements. Arch Oral Biol. 1961;5:301–22. Epub 1961/12/01. . 13976955 10.1016/0003-9969(61)90066-8

[14] Le Couteur DG, Solon-Biet S, Cogger VC, Mitchell SJ, Senior A, de Cabo R, et al The impact of low-protein high-carbohydrate diets on aging and lifespan. Cell Mol Life Sci. 2016;73(6):1237–52. Epub 2016/01/01. 10.1007/s00018-015-2120-y . 26718486 PMC11108352

[15] Simpson SJ, Le Couteur DG, James DE, George J, Gunton JE, Solon-Biet SM, et al The Geometric Framework for Nutrition as a tool in precision medicine. Nutr Healthy Aging. 2017;4(3):217–26. Epub 2017/12/26. 10.3233/NHA-170027 . 29276791 PMC5734128

[16] Raubenheimer D, Simpson SJ. Nutritional Ecology and Human Health. Annual Review of Nutrition, Vol 36. 2016;36:603–26. 10.1146/annurev-nutr-071715-051118 27296501

[17] Liang S, Hosur KB, Domon H, Hajishengallis G. Periodontal inflammation and bone loss in aged mice. J Periodontal Res. 2010;45(4):574–8. Epub 2010/03/27. 10.1111/j.1600-0765.2009.01245.x . 20337897 PMC2894296

[18] Reeves PG, Nielsen FH, Fahey GC Jr. AIN-93 purified diets for laboratory rodents: final report of the American Institute of Nutrition ad hoc writing committee on the reformulation of the AIN-76A rodent diet. J Nutr. 1993;123(11):1939–51. Epub 1993/11/01. 10.1093/jn/123.11.1939 8229312

[19] Andersson U, Rosen L, Wierup N, Ostman E, Bjorck I, Holm C. A low glycaemic diet improves oral glucose tolerance but has no effect on beta-cell function in C57BL/6J mice. Diabetes Obes Metab. 2010;12(11):976–82. Epub 2010/10/01. 10.1111/j.1463-1326.2010.01288.x . 20880344

[20] Solon-Biet SM, McMahon AC, Ballard JW, Ruohonen K, Wu LE, Cogger VC, et al The ratio of macronutrients, not caloric intake, dictates cardiometabolic health, aging, and longevity in ad libitum-fed mice. Cell Metab. 2014;19(3):418–30. Epub 2014/03/13. 10.1016/j.cmet.2014.02.009 . 24606899 PMC5087279

[21] Bolker BM, Brooks ME, Clark CJ, Geange SW, Poulsen JR, Stevens MH, et al Generalized linear mixed models: a practical guide for ecology and evolution. Trends Ecol Evol. 2009;24(3):127–35. Epub 2009/02/03. 10.1016/j.tree.2008.10.008 . 19185386

[22] Wood SN. Stable and efficient multiple smoothing parameter estimation for generalized additive models. J Am Stat Assoc. 2004;99(467):673–86. 10.1198/016214504000000980

[23] Wood SN. Fast stable restricted maximum likelihood and marginal likelihood estimation of semiparametric generalized linear models. J R Stat Soc B. 2011;73:3–36. 10.1111/j.1467-9868.2010.00749.x

[24] Wood SN. Thin plate regression splines. J R Stat Soc B. 2003;65:95–114. 10.1111/1467-9868.00374

[25] R-Development-Core-Team. R: A language and environment for statistical computing. Vienna: R Foundation for Statistical Computing; 2017.

[26] Bates D, Mächler M, Bolker B, Walker S. Fitting Linear Mixed-Effects Models Using lme4. Journal of Statistical Software. 2015;67(1).

[27] Mormann JE, Muhlemann HR. Oral starch degradation and its influence on acid production in human dental plaque. Caries Res. 1981;15(2):166–75. Epub 1981/01/01. 10.1159/000260514 . 6162561

[28] Bartold MP, Van Dyke TE. Host modulation: controlling the inflammation to control the infection. Periodontol 2000. 2017;75(1):317–29. Epub 2017/08/02. 10.1111/prd.12169 . 28758299

[29] Macia L, Tan J, Vieira A, Leach K, Stanley D, Luong S, et al Metabolite-sensing receptors GPR43 and GPR109A facilitate dietary fibre-induced gut homeostasis through regulation of the inflammasome. Nature communications. 2015;6(6734). Epub 1 April 2015. 10.1038/ncomms7734 25828455

[30] van der Velden U. The onset age of periodontal destruction. J Clin Periodontol. 1991;18(6):380–3. Epub 1991/07/01. . 1890216 10.1111/j.1600-051x.1991.tb02304.x

[31] Polak D, Wilensky A, Shapira L, Halabi A, Goldstein D, Weiss EI, et al Mouse model of experimental periodontitis induced by Porphyromonas gingivalis/Fusobacterium nucleatum infection: bone loss and host response. J Clin Periodontol. 2009;36(5):406–10. Epub 2009/05/08. 10.1111/j.1600-051X.2009.01393.x . 19419440

[32] Eke PI, Dye BA, Wei L, Slade GD, Thornton-Evans GO, Borgnakke WS, et al Update on Prevalence of Periodontitis in Adults in the United States: NHANES 2009 to 2012. J Periodontol. 2015;86(5):611–22. Epub 2015/02/18. 10.1902/jop.2015.140520 . 25688694 PMC4460825

[33] Trombone AP, Ferreira SB Jr., Raimundo FM, de Moura KC, Avila-Campos MJ, Silva JS, et al Experimental periodontitis in mice selected for maximal or minimal inflammatory reactions: increased inflammatory immune responsiveness drives increased alveolar bone loss without enhancing the control of periodontal infection. J Periodontal Res. 2009;44(4):443–51. Epub 2008/11/01. 10.1111/j.1600-0765.2008.01133.x . 18973535

[34] Napimoga MH, Clemente-Napimoga JT, Macedo CG, Freitas FF, Stipp RN, Pinho-Ribeiro FA, et al Quercetin inhibits inflammatory bone resorption in a mouse periodontitis model. J Nat Prod. 2013;76(12):2316–21. Epub 2013/11/20. 10.1021/np400691n . 24246038

[35] Lima HR, Gelani V, Fernandes AP, Gasparoto TH, Torres SA, Santos CF, et al The essential role of toll like receptor-4 in the control of Aggregatibacter actinomycetemcomitans infection in mice. J Clin Periodontol. 2010;37(3):248–54. Epub 2010/02/13. 10.1111/j.1600-051X.2009.01531.x . 20149215

